# Pseudo-ginsengenin DQ ameliorated aconitine-induced arrhythmias by influencing Ca^2+^ and K^+^ currents in ventricular myocytes†

**DOI:** 10.1039/d0ra01683g

**Published:** 2020-07-09

**Authors:** Lifang Jin, Cuizhu Wang, Jinping Liu, Pingya Li, Jing Li, Xiaoli Cui, Yi Wang

**Affiliations:** School of Pharmaceutical Sciences, Jilin University Fujin Road 126 Changchun 130021 China wangyijlu03@163.com +86-136-1072-9293; Department of Hematology and Oncology, The Second Hospital of Jilin University Changchun 130041 China; College of Basic Medical Sciences, Jilin University Changchun 130021 China

## Abstract

Pseudo-ginsengenin DQ (PDQ) is the product of the oxidative cyclization of protopanaxadiol. PDQ exhibits various bioactivities, including reversal of multidrug resistance in cancer, renal protective effects against acute nephrotoxicity and attenuating myocardial ischemia injury induced by isoproterenol or ligation of coronary arterials, but its effect on arrhythmias has not been clear until now. Because of the complicated effects of ginseng on the cardiovascular system, it is necessary to investigate whether PDQ affects arrhythmias, which are always concomitant with other cardiac diseases. Aconitine was used to induce arrhythmia *in vivo*. To understand its electrophysiological fundamental, whole-cell patch-clamp was used to record the L-type calcium current (*I*_Ca,L_) and potassium currents (*I*_K_ and *I*_K1_) in the ventricular myocytes in rats. Oral administration of PDQ exerted obvious antiarrhythmic effects, as indicated by the decreased incidence rate, lower number of occurrences, and shorter duration time of ventricular tachycardia and ventricular tachycardia, decreased mortality rate and increased survival time. *I*_Ca,L_ and *I*_K_ were inhibited by PDQ treatment while *I*_K1_ was not affected. To conclude, PDQ may have an anti-arrhythmia effect through inhibiting *I*_Ca,L_ and *I*_K_.

## Abstract 2

Pseudo-ginsengenin DQ (PDQ) is the product of the oxidative cyclization of protopanaxadiol. PDQ could ameliorate aconitine-induced arrhythmias by influencing Ca^2+^ and K^+^ currents in ventricular myocytes.

## Graphical abstract

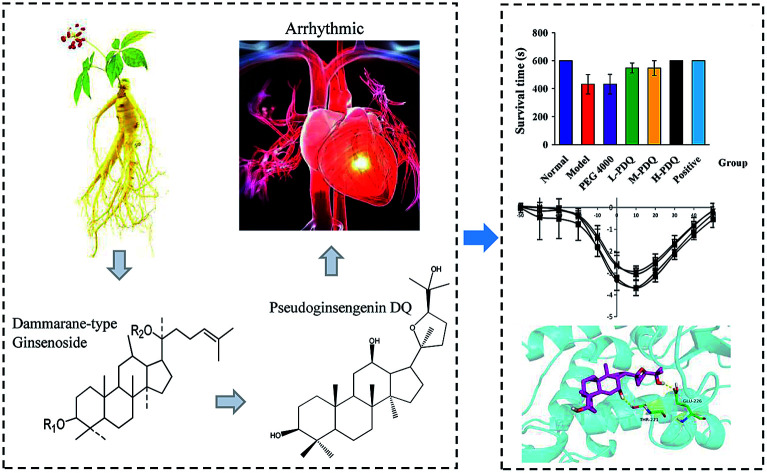

## 1. Introduction

Arrhythmias, as major types of heart malfunctions, often occur due to production or conduction of cardiac rhythm, which can cause hemodynamic changes, cardiac dilation, and ventricular filling pressure overload.^1–5^ Ginseng, the root of *Panax ginseng* Mayer, has been widely used to treat cardiovascular diseases in several countries.^6–9^ Ginsenosides are the main active ingredients, which were reported to have anti-arrhythmic effect.^10^ The main type of ginsenosides in ginseng is the dammarane type in the ginseng, while in the American ginseng it is the ocotillol-type (20,24-epoxyside)saponin. Ocotillol-type ginsenosides were reported to have multiple biological activities such as neuroprotective, anti-inflammatory, anti-bacterial, and anti-tumor effects.^11–14^ Therefore, some ocotillol-type saponins have been semi-synthesized by modifying dammarane-type saponins. Among them, Pseudo-ginsengenin DQ (PDQ) can be prepared by the oxidation and cyclization of the side-chain of protopanaxadiol with a yield of 75% (ZL 200510016774.4).^15^ PDQ has not only shown chemo-reversal ability in cancer *via* overcoming multidrug resistance,^16^ but also renal protective effects against CDDP-induced acute nephrotoxicity through the caspase and Sirt1/NF-κB signaling pathway.^17^ In addition, PDQ can attenuate isoproterenol-induced myocardial ischemia injury through suppressing the activity of glutathione peroxidase, reducing the superoxide dismutase and increasing the malondialdehyde content.^18^ The development of myocardial ischemia injury, along with the structural heart diseases and genetic deficits, has frequently been associated with arrhythmias.^19^ Thus, whether PDQ has anti-arrhythmic activity is necessary to be investigated.

Aconitine is a fatal toxin isolated from aconitum plant, which is the commonly used chemical to induce different kinds of arrhythmias, including ventricular premature beat (VP), ventricular tachycardia (VT), ventricular fibrillation (VF) and cardiac arrest, *etc.*^20^ Its electrophysiological mechanism involves mainly in multiple ion channels in myocyte membrane, such as activation of sodium channels to accelerate Na^+^ influx, depolarize cell membranes, and enhance the autonomy of fast-acting cells such as atrial conduction tissue and atrioventricular bundle-Purkinje fiber system, increasing Ca^2+^ inflow and inhibiting outward K^+^ current to prolong repolarization time, increase the incidence of post depolarization, and cause reentry excitation.

In the present study, PDQ pills were chosen as the test drug. PDQ pills were the dosage form with PDQ as drug substance and PEG 4000 (Polyethylene Glycol 4000) as excipients with a ratio of 1 : 3 in mass. We evaluated the anti-arrhythmic effects of PDQ pills on aconitine-induced arrhythmia in rats and investigated its electrophysiological mechanism of action in the cardiomyocytes of rat/guinea pigs *in vitro*.

## 2. Materials and methods

### 2.1 Chemicals and reagents

PDQ (purity > 98.0%) was provided by the School of Pharmaceutical Sciences of Jilin University (Changchun, China). PDQ pills (the dosage form) were prepared by Jilin Shengya Pharmaceutical Technology Co., Ltd. (Dunhua, China). PEG 4000 was bought from Hunan Erkang Pharmaceutical Co., Ltd. (Hunan, China). Chloral hydrate was purchased from Sinopharm Chemical Reagent Co., Ltd. Aconitine was bought from Hainan Sinochem United Pharmaceutical Industry Co., Ltd. Diltiazem hydrochloride tablets (catalogue number: 1009062, 30 mg per tablet) were purchased from Tianjin Tianbian Pharmaceutical Co., Ltd.

### 2.2 Animals and administration

#### 2.2.1 Animal

All the animal experiments were conducted based on the guide for the administration of laboratory animals (Directive 86/609/EEC in the Protection of Animals Used for Experimental and Other Scientific Purposes, 1986) and were approved by the Review Committee of Animal Care and Use of Jilin University. Male SPF Wistar rats weighing between 200 g and 220 g were purchased from the Animal Center of Norman Bethune Medical College of Jilin University (production certificate no. SCXK (JI) 2007-0003, SCXK (JI) 2013-0001; using certificate no. SCXK (JI) 2007-0011, SCXK (JI) 2013-0005). All rats had free access to standard laboratory food and water. They were maintained under standardized laboratory conditions (temperature 23 ± 1 °C, relative humidity 50–70%, 12 h light/dark cycle, with the light cycle beginning at 6:00 a.m., the ambient noise level was lower than 50 dB). *In vivo* experiment was carried out in rats and the rat ventricular myocytes were separated to investigate ion channel.

Guinea pigs weighing between 300 g and 350 g were purchased from the Animal Center of Norman Bethune Medical College of Jilin University (using certificate no. SCXK (JI) 2013-0005). Ventricular myocytes were separated to investigate action potentials.

#### 2.2.2 Preparation of arrhythmic model and PDQ pills administration

After a week of acclimatization, the rats were randomly divided into seven groups (*n* = 10 per group): (1) normal control group, (2) PEG 4000 group (240 mg kg^−1^), (3) aconitine-induced arrhythmic model group, (4) H-PDQ (high dose of PDQ pills, containing PDQ 80 mg kg^−1^), (5) M-PDQ (middle dose of PDQ pills, containing PDQ 40 mg kg^−1^), (6) L-PDQ (low dose of PDQ pills, containing PDQ 20 mg kg^−1^), (7) positive control group (diltiazem hydrochloride, 6 mg kg^−1^). The rats of normal and model groups were administrated 0.9% NaCl aqueous solution (5 mL kg^−1^). The rats of PEG 4000 group were administrated PEG 4000 (48.0 mg mL^−1^). PDQ groups were administered with PDQ pills aqueous solution (64.0, 32.0, 16.0 mg mL^−1^), which were grinded and suspended in 0.9% NaCl aqueous solution. The rats of positive drug group were administered with diltiazem hydrochloride aqueous solution (1.2 mg mL^−1^).

The rats in treatment groups were intragastrically administered with PEG 4000, PDQ or diltiazem hydrochloride 4 h before the experiment. Then, rats were anesthetized using 10% chloral hydrate. Aconitine (60 μg kg^−1^, 1 mL kg^−1^) was administrated *via* sublingual vein within 5 seconds, while the rats of normal group were injected with physiological saline.^21^ Electrocardiogram was recorded using a Power Lab Multi-channel physiological recorder (Power Lab15T, ML818, AD Instruments Shanghai Trading Co., Ltd., Shanghai, China).

### 2.3 Action potential (AP) measurement

Normal perfusion condition: the cells were divided into DMSO (0.1%) perfused control group and different concentrations of PDQ (0.2 μg mL^−1^, 2.0 μg mL^−1^, 20.0 μg mL^−1^).

### 2.4 Solutions preparation

Ca^2+^ Tyrode's solution contained (in mM): NaCl 143.0, KCl 5.4, CaCl_2_·2H_2_O 1.8, MgCl_2_·6H_2_O 1.8, NaH_2_PO_4_·2H_2_O 0.25 and HEPES 5.0 (adjusted to pH 7.4 with NaOH). The Ca^2+^-free Tyrode's solution was prepared by removing CaCl_2_ from the Tyrode's solution. The extracellular solution contained (in mM): NaCl 115.0, KCl 4.7, MgSO_4_ 1.2, CaCl_2_·2H_2_O 2.0, NaHCO_3_ 2.5, KH_2_PO_4_ 1.2, glucose 10.0, tetrodotoxin (TTX) 20.0, 4-aminopyridine (4-AP) 5.0 (pH 7.4 adjusted with NaOH). The pipette solution for recording *I*_Ca,L_ and *I*_K1_ (the inward rectifier potassium current) contained (in mM): cesium aspartate 120.0, CsCl 30.0, KH_2_PO_4_ 10.0, MgSO_4_ 3.0, NaCl 5.0, HEPES 5.0, K_2_ATP 5.0, EGTA 0.1 (pH 7.3 adjusted with CsOH). The pipette solution for recording *I*_K_ (outward delay rectifier potassium current) contained (in mM): potassium aspartate 80.0, KCl 42.0, KH_2_PO_4_ 10.0, Mg^2+^-ATP 10.0, phosphocreatin 3.0, K^+^-EGTA 5.0 (pH 7.2–7.4 adjusted with KOH). The Kreb's solution used for cell storage contained (in mM): KOH 70.0, l-glutamic acid 50.0, KCl 40.0, taurine 20.0, KH_2_PO_4_ 20.0, MgCl_2_·6H_2_O 3.0, glucose 10.0, HEPES 10.0, EGTA 0.5 (pH 7.4 adjusted with KOH).

PDQ was dissolved in 0.1% DMSO, the final concentration was 200 μg mL^−1^.

### 2.5 Myocyte preparation

Wistar rats/adult guinea pigs were anesthetized with sodium phenobarbital (30 mg kg^−1^, intraperitoneally), and the heart was excised and fixed in a Langendorff apparatus. The Ca^2+^ Tyrode's solution was used to retrograde perfusion in the heart for the first 2 min, then the Ca^2+^-free Tyrode's solution for 5 min, and at last the enzymatic solution (15 mg collagenase in 100 mL Ca^2+^-free Tyrode's solution) for 10 min. Afterwards, the ventricular muscle were cut into small pieces (1 mm^3^) and lightly oscillated for 10 min at 37 °C. Ventricular myocytes were filtered through nylon mesh and stored in Krebs solution at 4 °C. The perfusate was bubbled with 95% O_2_ + 5% CO_2_ at a flow of 8 mL min^−1^ at 37 °C. Ventricular myocytes from Wistar rats were used to measure the L-type calcium currents, while the myocytes of adult guinea pigs were used to measure the action potential of ventricular myocytes and the potassium currents.

### 2.6 Whole-cell patch-clamp recording

Isolated single ventricular myocytes were used for the whole-cell recordings. Glass microelectrodes were constructed by a two-stage pulling and then the electrode resistance was maintained at 1.0–3.0 MΩ when filled with the internal electrode solution. L-type calcium currents, and potassium currents were recorded with a CEZ-2400 amplifier (Nihon Kohden, Japan) and linked to a 12 bit analog to digital converter (AD/DA) card (Axon, America) at 3 KHz. pCLAMP 6.03 software (Axon Instruments) was applied to generate voltage clamp protocols and retrieve data. Clampfit 6.02 software (Molecular Devices, America) was used to analyze the current traces.

### 2.7 Molecular docking of PDQ

To clarify the mode of action of PDQ on *I*_Ca,L_ and *I*_K_, a molecular docking study was carried out to measure the relative binding energies and localized binding sites in the active pocket. The study was performed using GLIDE (Grid-based Ligand Docking with Energetics) (GLIDE, version 6.7, Schrödinger, LLC, New York, USA, 2015) software developed by Schrödinger. Maestro Elements (2015-2) was used for all the steps involving protein and ligand preparation, receptor grid generation, and docking. The X-ray crystal structure of calcium channel [Protein Data Bank (PDB) code: 4TMF] and potassium channel (PDB code: 2A79) were retrieved from the PDB database (http://www.rcsb.org/pdb) according to previous studies.^22,23^ The Protein Preparation Wizard in the GLIDE software was used to prepare the receptors. The structures of proteins were optimized after a series of processes, including assigning bond orders and water orientations, removing water, adding hydrogen, and creating zero-order bonds to metals and di-sulphide bonds.^24^ Two-dimensional structure of PDQ (ligand) were drawn with Maestro Elements (Maestro Elements, 2.2). LigPrep module (2015-2) of Schrödinger Suite was used to generate the three-dimensional structure of PDQ by assigning the bond orders and angles. In addition, the ligand was subjected to minimization using the OPLS3 force field. For GLIDE docking, the prepared structure of calcium channel, potassium channel, and ligand (PDQ) were imported into the workspace using GLIDE v6.7 from Schrödinger Suite.^25–27^ Extra precision (XP) docking was carried out, and the parameters of scaling factor and partial charge cutoff were set at the default values 0.80 and 0.15, respectively.^28^ Figures of the docking results were subsequently prepared using PyMOL (Schrödinger).

## 3. Statistical analysis

All data are expressed as the mean ± standard deviation. GraphPad Prism 7.0 software was applied to analyze the data. Data analyses were performed using one way ANOVA. *p* < 0.05 was considered statistically significant.

## 4. Results

### 4.1 PDQ reduced aconitine-induced arrhythmias and mortality in rats in dose-dependent manner

**Fig. 1:**
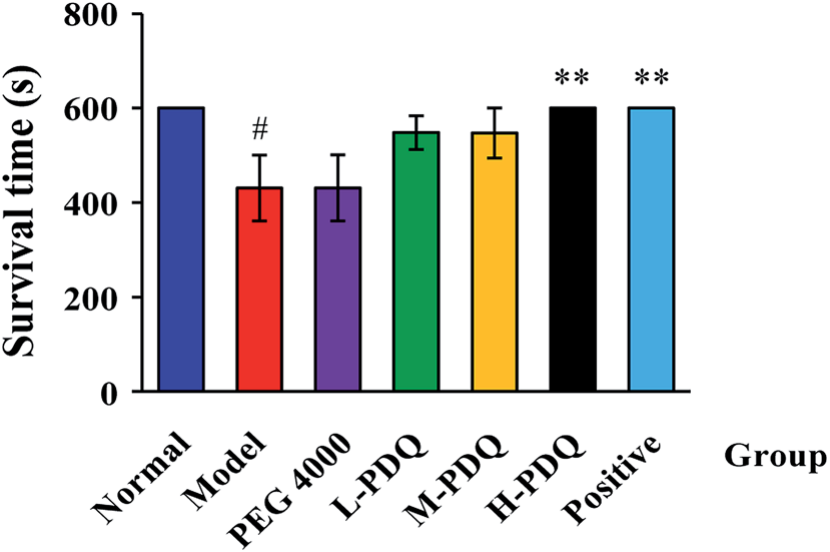
Effects of PDQ on survival time of aconitine-induced arrhythmias (compared with normal group, ^#^*p* < 0.05; compared with model group, **p < 0.01).

**Fig. 2:**
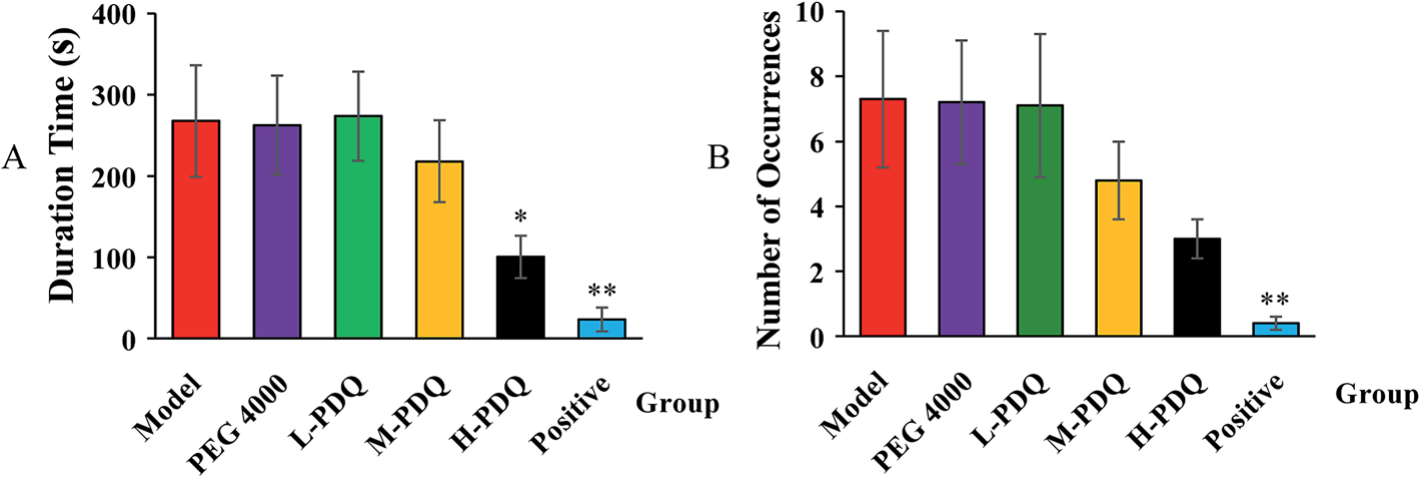
Effects of PDQ on duration time (A) and number of occurrences (B) of ventricular tachycardia (VT) (compared with model group, *p < 0.05, **p < 0.01).

Compared with the normal control group, the significantly elevated incidence rate of VT and VF were 100% and 60% respectively in the aconitine-induced group, suggesting the chemical induced arrhythmic model was successfully established. The PEG 4000 group showed no effect on the model rats. The mortality rate of aconitine-induced group (Model) was 40%, while the mortality rates of low-, medium- and high-doses of PDQ (L-PDQ, M-PDQ and H-PDQ) were 20%, 10%, and 0% respectively (Table 1). In addition, aconitine-induced group showed a significantly shorter survival time (*p* < 0.01). As for both of the positive control group and H-PDQ group, the survival time were 600 s, which showed significantly difference compared with model group (Fig. 1). Moreover, the H-PDQ showed a significantly decreased arrhythmic incidence and shorter duration time of VT (*p* < 0.01), which was similar to the positive group (Table 1 and Fig. 2). PDQ reduced aconitine-induced arrhythmias and mortality in rats in dose-dependent manner.

**Table 1:** The mortality rate, VT incidence and VF incidence^a^

Group	Mortality rate (%)	VT incidence (%)	VF incidence (%)
Normal	0.0	0.0	0.0
PEG 4000	40.0	100.0	60.0
Model	40.0^#^	100.0^##^	60.0^##^
L-PDQ	20.0	100.0	30.0
M-PDQ	10.0	100.0	10.0*
H-PDQ	0.0**	90.0	0.0**
Positive	0.0**	30.0**	20.0

a Compared with normal group, ^#^*p* < 0.05, ^##^*p* < 0.01; compared with model group, **p < 0.01.

### 4.2 PDQ prolonged APD and reduced APA in ventricular myocytes

PDQ pre-treatment has the tendency of prolonging action potential duration (APD) with the increase of dosage. There is no significant difference for APD at 50% repolarisation (APD_50_) between PDQ treated groups and normal group. However, 2.0 μg mL^−1^ (*p* < 0.05) and 20.0 μg mL^−1^ PDQ (*p* < 0.01) significantly prolonged the APD at 90% repolarization (APD_90_). In addition, 20.0 μg mL^−1^ PDQ remarkably reduced the action potential amplitude (APA) (*p* < 0.001) *versus* the control group. The absolute values of resting potential (RP) were significantly reduced by 2.0 μg mL^−1^ PDQ and 20.0 μg mL^−1^ PDQ treatment. The results were shown in Table 2. These results suggested that PDQ treatment may probably influence the phase 3 or 4 of APD, but the detail mechanisms need to be investigated.

**Table 2:** Effects of PDQ on action potential in rat ventricular myocytes *in vitro* (±s)^a^

Group	Dose (μg mL^−1^)	APA (mV)	*V*_max_ (V s^−1^)	RP (mV)	APD50 (ms)	APD90 (ms)
Normal	—	107.96 ± 5.73	13.49 ± 1.40	−82.85 ± 4.32	253.65 ± 42.23	296.52 ± 39.23
PDQ-1	0.002	107.11 ± 5.71	13.87 ± 1.79	−82.44 ± 5.95	251.80 ± 49.93	296.58 ± 44.97
PDQ-2	0.02	108.45 ± 5.13	12.21 ± 0.90	−82.06 ± 7.78	265.15 ± 49.52	310.88 ± 41.68
PDQ-3	0.2	106.37 ± 2.02	12.97 ± 1.56	−82.53 ± 2.63	277.57 ± 48.83	327.35 ± 39.41
PDQ-4	2.0	104.39 ± 10.96	13.87 ± 2.75	−74.07 ± 3.46**	291.77 ± 47.20	346.32 ± 37.29*
PDQ-5	20.0	91.10 ± 2.48***	12.36 ± 2.61	−69.75 ± 4.78***	315.00 ± 53.56	364.40 ± 40.74**

a Compared with normal group, *p < 0.05, **p < 0.01, ***p < 0.001.

### 4.3 PDQ reduced L-type calcium peak current in dose-dependent pattern

**Fig. 3:**
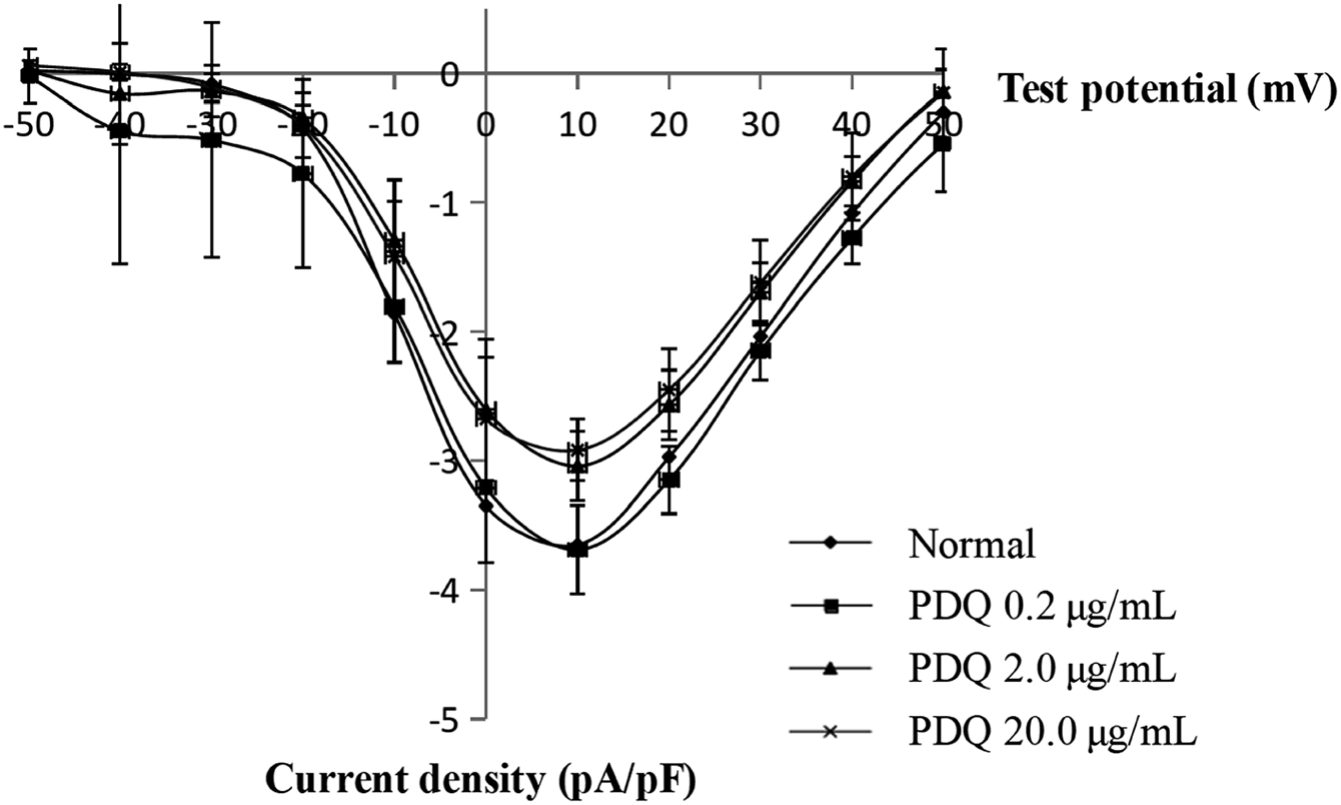
Effects of PDQ on current–voltage curve of *I*_Ca,L_ in different groups.

In the normal perfusion condition, *I*_Ca,L_ was elicited by a depolarizing pulse to −40 mV from a prepulse of +10 mV (holding potential −50 mV) in ventricular cells. Flip potential was at +50 mV to +60 mV, the maximum *I*_Ca,L_ (*I*_Ca,L_, peak) was −3.64 ± 0.35 pA pF^−1^. The current was sensitive to verapamil, indicating that it was L-type Ca^2+^ current. The average maximum *I*_Ca,L_ of 0.2, 2.0 and 20.0 μg mL^−1^ PDQ were −3.64 ± 0.34 pA pF^−1^, −3.03 ± 0.27 pA pF^−1^ (*p* < 0.05) and −2.91 ± 0.24 pA pF^−1^ (*p* < 0.01) *vs.* −3.64 ± 0.35 pA pF^−1^ in control group (Fig. 3), indicating that PDQ exerted anti-*I*_Ca,L_ effect in a dose-dependent manner.

### 4.4 PDQ inhibited delayed rectifier K^+^ channels (*I*_K_), not inward rectifier K^+^ channels (*I*_K1_)

**Fig. 4:**
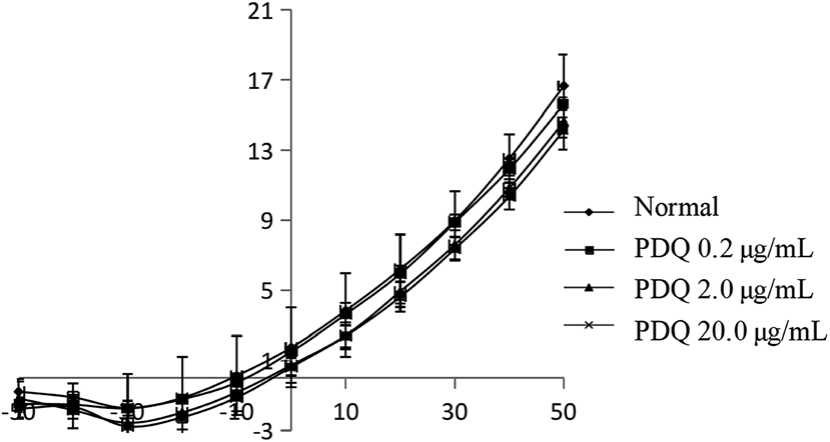
Effects of PDQ on current–voltage curve of *I*_K_.

In common condition, *I*_K_ was from −50 mV to +50 mV by a prepulse of +10 mV (holding potential −40 mV) in ventricular cells within 400 ms. In the present study, *I*_K_ was activated at −30 mV in the normal group, and the maximum peak outward current was 16.67 ± 1.78 pA pF^−1^ at +50 mV. After adding tetraethylammonium (TEA), the potential amplitude was inhibited, indicating it was *I*_K_ current. After perfusion for 3 min using different concentrations of PDQ, the peak current at +50 mV of 0.2, 2.0 and 20.0 μg mL^−1^ PDQ were 15.63 ± 0.36 pA pF^−1^, 14.63 ± 0.92 pA pF^−1^ (*p* < 0.05) and 14.15 ± 1.12 pA pF^−1^ (*p* < 0.05) *vs.* 16.67 ± 1.78 pA pF^−1^ in control group. Current–voltage curve moved downward, suggesting that PDQ can inhibit *I*_K_ in a dose-dependent pattern (Fig. 4).

### 4.5 PDQ has no effect on *I*_K1_ in ventricular myocytes

**Fig. 5:**
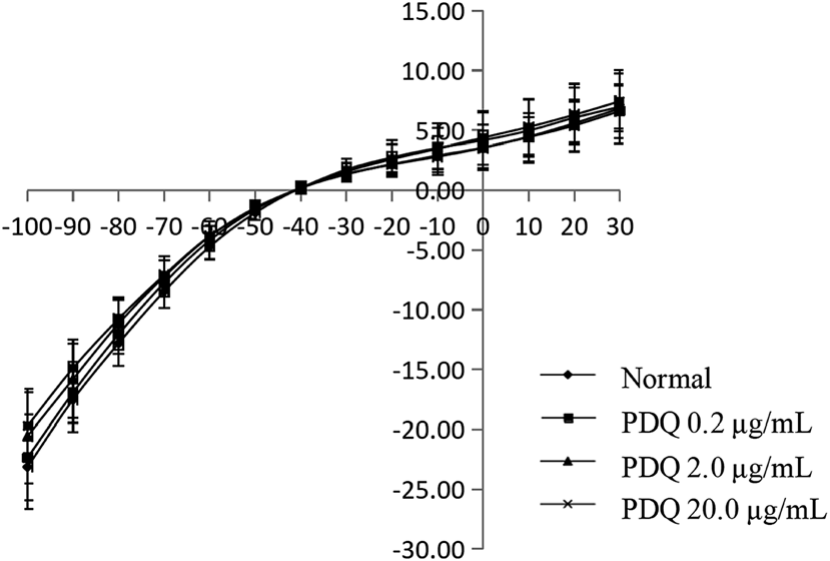
Effects of PDQ on current–voltage curve of *I*_K1_.

In the normal perfusion condition, *I*_K1_ was elicited by a depolarizing pulse to +30 mV from a prepulse of −100 mV (holding potential −40 mV) in 10 mV increments in ventricular cells. Then it reached a steady state, and didn't change over time, showing inward rectification characteristics. Flip potential was about −40 mV, and the maximum *I*_K1_ was −23.12 ± 3.55 pA pF^−1^ at −100 mV. The *I*_K1_ of 0.2, 2.0 and 20.0 μg mL^−1^ PDQ were −22.35 ± 3.58 pA pF^−1^, −20.55 ± 3.94 pA pF^−1^ and −19.71 ± 2.80 pA pF^−1^, which showed no significant difference compared with the normal group, indicating PDQ had no effect on *I*_K1_ (Fig. 5).

### 4.6 Molecular docking for calcium and potassium channel

**Fig. 6:**
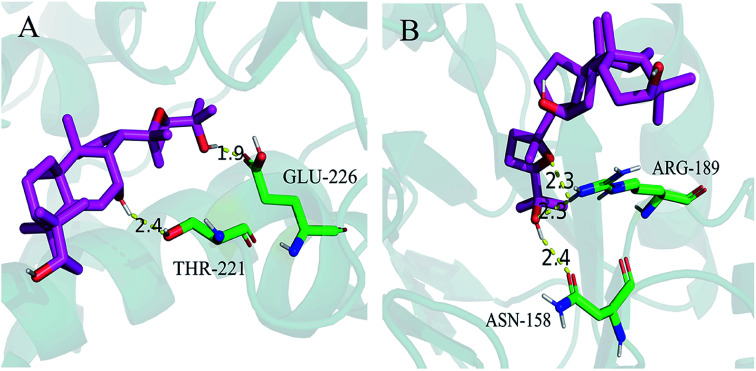
Docked poses of PDQ in 4TMF (A) and 2A79 (B).

Molecular docking was used to measure the localized binding sites and relative binding energies in the active pocket. As displayed in Fig. 6A, the model provided the most probable binding sites and structural configurations of PDQ in calcium channel (4TMF). PDQ formed one hydrogen bond at a distance of 1.9 Å with the residue (GLU-226), and 2.4 Å with the residue (THR-221). Docking calculation predicted three hydrogen bonds forming between PDQ and ASN-158 and ARG-189 of the potassium channel (2A79, Fig. 6B), two hydrogen bond at a distance of 2.3 Å with the residue (ARG-189), and one hydrogen bond with the residue (ASN-158) at a distance of 2.4 Å identified in the binding pocket. This result was consistent with the electrophysiological result for calcium and potassium.

## 5. Discussion

PDQ belongs to ocotillol type saponogenin, a type that was first found in *Panax quinquefolium*, and is an important and symbolic component of *Panax quinquefolium*. In terms of the complicated effects of ginseng on inotropic and chronotropic effect of the cardiovascular system, it is necessary to clarify the influence of PDQ on different ion channels which are the electrophysiological base for various arrhythmias. In our experiment *in vitro*, PDQ pre-treatment significantly prolonged APD90 in ventricular myocytes of guinea pigs in a concentration-dependent manner, but the APD50 prolongation has no significant difference compared with the normal group. These observations indicated that PDQ may change the ions flow in phase 2 or 3 which are involved in the Ca^2+^ and K^+^ ion influx and efflux cell membrane.

Calcium channels include mainly T type and L type. L-type calcium channels are the most import one for their long duration time, high activation voltage and large conductance. *I*_Ca,L_ is the major pathway for facilitating the influx of calcium into cells when cells were excited. *I*_Ca,L_ also plays an important role in cardiac excitability-contraction coupling, the self-discipline of cardiac sinus node and the conduction of atrioventricular node. Nowadays, most calcium channel blockers are targeted at *I*_Ca,L_. In this study, the potential was set at −40 mV, thus it can not only exclude the interference from T-type calcium channels but also avoiding the effects of sodium current. K^+^ was used instead of Ca^2+^, resulting in block of the potassium current in cardiomyocytes. There was almost no current depletion because data was collected within 10 min, which guaranteed the reliability of results. 2.0 μg mL^−1^ PDQ and 20.0 μg mL^−1^ PDQ inhibited *I*_Ca,L_ under different membrane potentials, indicating that PDQ suppressed voltage-dependent *I*_Ca,L_, which may result in the prolonged APD. *I*_K_ participated in the repolarization process of AP, which played an important role in regulating both the termination of action potential in 2-phase plateau and 3-phase repolarization. The inhibition of *I*_K_ by PDQ in dose-independent manner suggested *I*_K_ was the major current for prolonging APD. Moreover, PDQ had no effect on *I*_K1_, which was closely relevant for RP. In a word, PDQ may exerted anti-arrhythmic effect by inhibiting *I*_Ca,L_ and *I*_K_ together. The molecular docking study also verified that PDQ has the relative binding energies and localized binding sites for calcium channel and potassium channel, which was consistent with the results of the animal experiment. Moreover, RP is important for maintaining the excitability of ventricular myocytes. The reduced RP by 2.0 μg mL^−1^ PDQ and 20.0 μg mL^−1^ PDQ treatment shortened the distance from the threshold potential, so the excitability of ventricular myocytes was elevated considering the stable *V*_max_.

Aconitine can lead to excitation of cardiac vagus nerve, which can reduce the autonomy of sinoatrial node, excite the ectopic rhythm point in the ventricle, and lead to multiple reentry in the process of cardiac repolarization. Many studies have reported that aconitine had non-selectivity for Na^+^, Ca^2+^ and K^+^ ion channel. Therefore, PDQ anti-arrhythmic effect by aconitine-induced in rats is likely the integrated effect resulting from improved cardiac repolarization of Ca^2+^ and K^+^. The prolongation of APD is the main reason for terminating reentrant arrhythmias.

Owing to that APD was regulated by multiple ion channels in membrane. *I*_Ca,L_ current was critical to the depolarization and repolarization of AP while *I*_K_ was the major current in phase 3 of repolarization. Therefore, the APD and the effective refractory period were regulated by *I*_Ca,L_ and *I*_K_ together. In addition, *I*_K1_, the dominant current of RP, can affect the excitability of ventricular myocytes by regulating RP. In terms of electrophysiology, *I*_Ca,L_ and *I*_K_ changes seem to be more important in revealing the mechanisms of anti-arrhythmic effect of PDQ.

## Author contributions

Investigation, Writing-original draft, L. J.; Software, C. W.; Methodology, X. C. & J. Li.; Conceptualization, Y. W.; Funding acquisition, P. L.; Writing-review and editing, J. Liu.

## Fundings

This research was supported by the Science and Technology Development Plan of Jilin Province (No. 201603033YY) and Key Technologies Research and Development Program (No. 2017YFC1702105).

## Conflicts of interest

The authors declare that they have no conflict of interest concerning this article.

## Supplementary Material

RA-010-D0RA01683G-s001

RA-010-D0RA01683G-s002

RA-010-D0RA01683G-s003

RA-010-D0RA01683G-s004
